# Gold nanowire mesh electrode for electromechanical device

**DOI:** 10.1038/s41598-023-43960-8

**Published:** 2023-10-04

**Authors:** Taichi Ikeda

**Affiliations:** Research Center for Macromolecules and Biomaterials, National Institute for Materials Science, 1-1 Namiki, Tsukuba, Ibaraki 305-0044 Japan

**Keywords:** Chemistry, Engineering, Materials science, Nanoscience and technology

## Abstract

Ionic polymer-metal composite (IPMC) actuators were prepared with Nafion film as the ionic polymer and gold nanowire (Au-NW) mesh film as the metal electrodes by hot-pressing, which shortened preparation time within 1 h. As a reference, IPMC actuator consisting of Nafion film and gold foil (Au-foil) was also prepared. Au-NW mesh film can be an electrode with thinner (about 150 nm) and lower surface resistivity (about 0.5 Ω sq^−1^) than the conventional electrode prepared by electroless plating. Larger contact area of the Au-NW mesh electrode than the Au-foil electrode resulted in better actuation performance (60% larger peak-to-peak displacement in actuation). It was confirmed that the transformation behavior of Au-NWs differed depending on the external stimuli condition. Namely Au-NWs transformed to Au nanoparticles in the case of the heat stimulus only. Meanwhile, Au-NWs transformed to plates in the case of the heat and pressure stimuli. While higher temperature improved the adhesion of Au-NW mesh electrode to the Nafion surface, it induced the transformation of nanowire to plates. The IPMC actuator that the Au-NW mesh electrodes were hot-pressed at 90 ºC exhibited the highest capacitance and the largest peak-to-peak displacement in actuation. This research expanded the application field of gold nanowires to the electromechanical devices.

## Introduction

Ionic polymer-metal composite (IPMC) actuator, which consists of an ionic polymer sandwiched by the metal electrodes, is one of the most popular actuators in the fields of biomedical devices, flexible robots and microelectromechanical systems because of some advantages, light-weight, simple configuration, low operation voltage (< 5 V) and silent bending motion etc^1–5^. The typical combination of ionic polymer and electrode is a sulfonated tetrafluoroethylene polymer called “Nafion” and a noble metal electrode such as gold or platinum, respectively^6,7^. In most cases, the noble metal electrodes have been prepared on the Nafion surface by electroless plating^2,4,8^. It takes several days to prepare the IPMC actuator, because the electroless plating requires to repeat the following two processes. (i) Nafion film is immersed in the metal ion solution for 1 day. (ii) Nafion film is immersed in the reduction solution for 1 day. On the other hand, hot-pressing is popular to attach the conducting carbon film on the ionic polymer because of simple and quick process^9–11^. The hot-pressing method has not been applied to attach the thin noble metal electrodes on the Nafion surface. J. H. Park reported the nano-dispersed metal electrodes improved the performance of IPMC actuator because of large contact area of the electrode to the Nafion surface^12^. On the basis of this background, the objective of this study is to tackle the following two issues. The first issue is to confirm whether hot-pressing is available to attach the gold nanowire (Au-NW) mesh film and the thin gold foil (Au-foil) on the Nafion surface as the electrodes. If possible, one can drastically shorten the preparation time of the IPMC actuator. The second issue is to confirm that the Au-NW mesh electrode gives better actuation performance than the Au-foil electrode because of larger contact area to the Nafion surface. Although some researchers utilized silver (Ag) or copper nanowires for IPMC actuators^13,14^, these nanowires are easily oxidized by (electro)chemical reaction. Au-NW, which has excellent chemical stability^15–17^, is suitable for the electrode material of IPMC actuators. Y. Wang reported rapid preparation of IPMC humidity sensor with Au-shell-Ag-NW by spraying and electrodepositing^15^. Recently, some research groups started to report the application of Au-NWs to electronic and sensor devices^18,19^. However, the application fields of Au-NWs are still limited. Here, it was confirmed that Au-NW mesh electrode could be a promising electrode material for IPMC actuators, which will expand the application field of Au-NWs to the electromechanical devices.

## Materials and methods

### Materials and instruments

Nafion 117, hydrophilic polytetrafluoroethylene (PTFE) filter Omnipore (pore size: 1.0 μm, diameter: 47 mm), highly ordered pyrolytic graphite (HOPG) and hydrogen tetrachloroaurate(III) trihydrate (HAu(III)Cl_4_·3H_2_O) were purchased from Merck. Sodium borohydride (NaBH_4_), ascorbic acid and sulfuric acid (95%) were purchased from Tokyo Chemical Industry. Trisodium citrate dihydrate, cetyltrimethylammonium bromide (CTAB), hydrochloric acid (35%), hydrogen peroxide (35%) and distilled water were purchased from Nacalai Tesque. Au-foil (Gold leaf, Au: 99.99%) was purchased from Kanazawa Katani. Kapton film was purchased from Du Pont-Toray Co.

IPMC actuators were prepared with two press machines Mini Test Press MP-SCL and MP-SCH (Toyoseiki). Scanning electron microscopy (SEM) measurement was conducted on a Hitachi S-4800 field emission scanning electron microscope (accelerating voltage: 5–20 kV). A cross-section samples of IPMC actuators were prepared using freeze fracturing in liquid nitrogen. The sample surface was coated with carbon prior to SEM measurement. The surface resistivity was measured with a resistivity meter Loresta-GX MCP-T700 (Nittoseiko Analytech). The capacitance was measured using two-terminal impedance spectroscopy on a Solartron SI 1260 system with a 1296 Dielectric Interface. The sample was placed on a disk-type blocking electrode of a Solartron 12962A dielectric sample holder. The frequency was swept from 100 to 0.1 Hz by applying an AC voltage of 10 mV and a DV bias of 0.1 V. From the imaginary part of the measured impedance, the differential double-layer capacitance was determined by the following Eq. (1):^20^

1 $${C}_{\mathrm{d}}\left(f\right)=\frac{-1}{2\pi fZ"\left(f\right)}$$

where *C*_d_(*f*), *f* and *Z"*(*f*) are differential double-layer capacitance, frequency and imaginary part of the measured impedance, respectively.

For actuation measurement, the applied voltage was controlled by a function generator DG812 (RIGOL). The actuation behavior was monitored by a laser displacement sensor HL-G108-S-J (Panasonic). The data was recorded with a data acquisition recorder MR8870 (Hioki).

### Preparation of Au-NW

Au-NW was prepared according to the literatures^21–23^. The protocol is described briefly. First, the gold nanoparticle was prepared. 10 mL of HAuCl_4_ aqueous solution (0.5 mM) and 10 mL of trisodium citrate aqueous solution (0.5 mM) were mixed in a round bottom flask. 0.6 mL of ice cold NaBH_4_ aqueous solution (0.1 M) was added to the gold ion solution with vigorous stirring (1000 rpm) at room temperature. The color of the solution turned red immediately which indicates the formation of gold nanoparticles. Second, the gold nanorod was prepared. CTAB (21.9 g) was dissolved in distilled water (594 mL). HAuCl_4_ (0.05 M, 3 mL) and ascorbic acid (0.1 M, 3 mL) aqueous solutions were added to the CTAB solution sequentially. The solution was separated into three (Solution 1: 15 mL in a 20 mL vial; Solution 2: 45 mL in a 50 mL vial; Solution 3: 540 mL in a 1 L conical flask). 1 mL of gold nanoparticle solution was added to the Solution 1. Within 5 s, 4.5 mL of the Solution 1 was added to the Solution 2. Within 5 s, all of the Solution 2 was added to the Solution 3. The solution was kept undisturbed in an oven at 27 °C for 40 h. The supernatant of the solution was removed by decantation. The precipitate was recovered with CTAB aqueous solution (0.1 M, 50 mL). The dissolution solution was prepared by dissolving CTAB (0.1 M) and HAuCl_4_ (0.5 mM) in distilled water. In order to remove impurities, 5 mL of the dissolution solution was added to the suspension of the recovered precipitate in the CTAB solution (0.1 M, 50 mL). The solution was kept undisturbed in an oven at 35 °C for 12 h. The blue-colored supernatant was removed by decantation. The dissolution process of the impurities was done in four times. The gold nanorod was dispersed in 20 mL of 0.1 M CTAB solution. At last, the Au-NW was prepared. For 1D growth of gold nanorods, the growth solution was prepared by sequential addition of HAuCl_4_ (0.25 mM, 0.5 mL), HCl (35%, 1.0 mL) and ascorbic acid (0.1 M, 0.5 mL) aqueous solutions to the CTAB solution (0.1 M, 98 mL). 0.5 mL of the gold nanorod solution was added to the growth solution (100 mL). The solution was kept undisturbed in an oven at 27 ºC for 24 h. The Au-NW was obtained as a brown-colored precipitate.

### Preparation of Au-NW mesh film

The Au-NWs in the CTAB aqueous solution prepared above (100 mL) were re-dispersed and vacuum-filtrated through a PTFE filter (pore size: 1.0 μm, diameter: 47 mm). The film was washed with distilled water for removing CTAB, then dried in a vacuum oven at room temperature. The weight of the Au-NW mesh film was obtained by subtracting the PTFE filter weight from the total weight of the filter and Au-NW mesh film. The length and width of Au-NW were measured from the SEM images (Fig. S1) with the software ImageJ Ver. 1.53 k (http://imagej.nih.gov/ij). The collected data (170 data points) were summarized in the histograms of Fig. S2.

### Preparation of IPMC actuator

Nafion film was treated in an aqueous solution of hydrogen peroxide (5%) at 80 ºC for 1 h, sulfuric acid (5%) at 100 °C for 1 h, and distilled water at 100 °C for 1 h. Then the Nafion film was dried in a vacuum oven. The Nafion was cut into the pieces with the dimension of 15 mm × 15 mm. The Nafion film was sandwiched by the Au-NW mesh films between the stainless-steel plates with a spacer (Fig. S3). The assembled stainless-steel plates were set to the hot-pressing machine. They were pressed at 5 MPa at room temperature for 1 min, then at 3 MPa at a setting temperature (refer as preparation temperature) for 10 min. After flipping the assembled plates, they were pressed at 3 MPa at a setting temperature for another 10 min, then pressed at 3 MPa at room temperature for 20 min. Finally, the PTFE filter was peeled off. After the transfer of the Au-NW mesh film on a Nafion surface, the Au-NW mesh film is referred as Au-NW mesh electrode. The Nafion film sandwiched by the Au-NW mesh electrodes was cut into the pieces with the dimension of 3 mm width and 15 mm long. The IPMC actuator consisting of the Au-foil electrodes was prepared in the same procedure utilizing the Nafion film and Au-foil.

## Results and discussion

### Characterization of Au-NW mesh film

**Figure 1:**
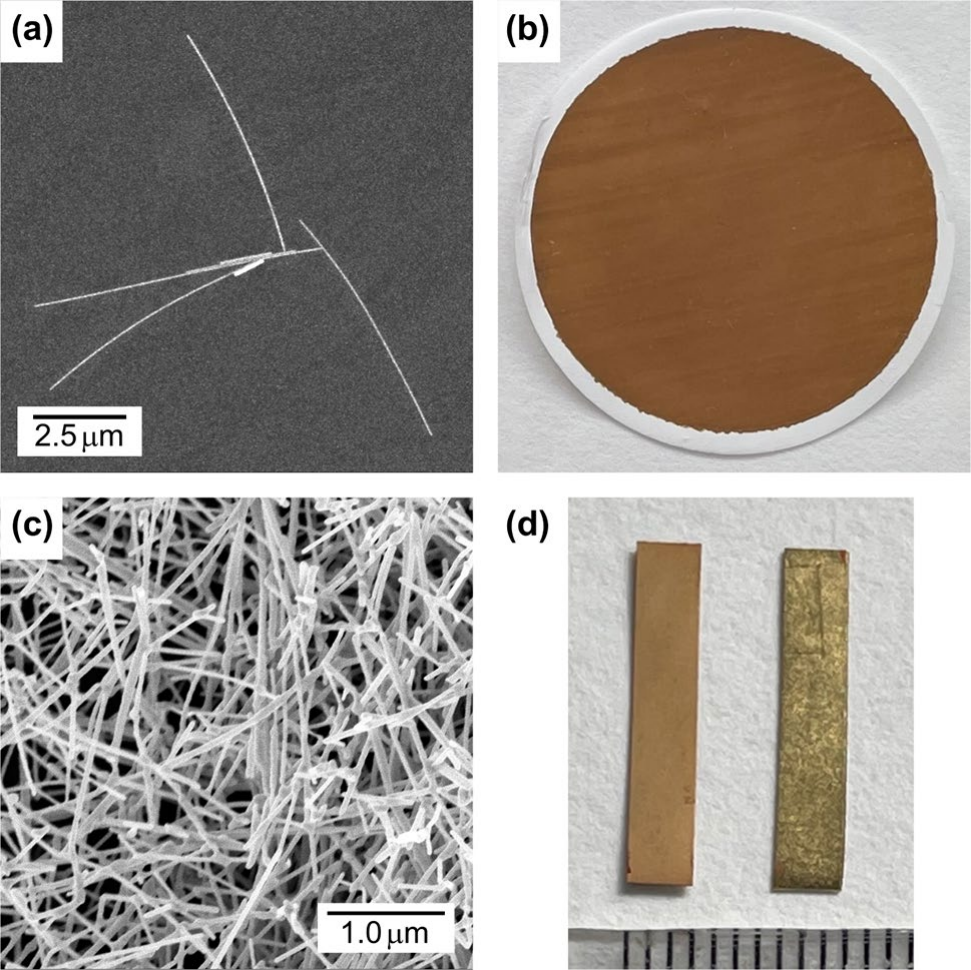
Characterization of Au-NW and Au-NW mesh film. (**a**) SEM image of Au-NWs on a HOPG substrate. (**b**) Photograph of Au-NW mesh film on a membrane filter. (**c**) SEM image of Au-NW mesh film. (**d**) IPMC actuator strips. Left: Au-NW mesh electrode. Right: Au-foil electrode.

Figure 1a shows the SEM image of Au-NWs on a HOPG substrate. The average length and width of Au-NW were 6.3 ± 2.9 μm and 43.2 ± 8.5 nm, respectively (average ± standard deviation, *n* = 170, Fig. S2). Au-NW mesh film was prepared by vacuum filtration of the Au-NW dispersion solution through a membrane filter. The color of the Au-NW mesh film is brown (Fig. 1b) because of the characteristic absorption peak of surface plasmon resonance^23–25^. The SEM image of the Au-NW mesh film proved little impurities such as Au nanoparticle and nanoplatelet. (Fig. 1c). When the purification process of Au nanorod was not perfect, Au nanoplatelets were contaminated. The weight of the Au-NW mesh film was 4.2 ± 0.2 mg (Average ± standard deviation, *n* = 5). The surface resistivity was 1.23 ± 0.24 Ω sq^−1^ (Average ± standard deviation, *n* = 5).

### Preparation of IPMC actuators

The preparation process of IPMC actuators is schematically illustrated in Fig. S3. The Au-NW mesh films were hot-pressed on the both sides of the Nafion film as the electrodes. When the membrane filter was peeled off, the Au-NW mesh film was completely transferred on the Nafion surface, supporting good adhesion of Au-NW mesh film on the Nafion surface. In the hot-pressing process, the spacer is indispensable. When hot-pressing was done without a spacer, the Nafion film stretched laterally by compression, which caused the cracks in the Au-NW mesh electrode and resulted in high surface resistivity. Four IPMC actuators with the Au-NW mesh electrodes were prepared at different temperatures (Sample #1–4 in Table 1). It is expected that higher hot-pressing temperature is advantage for softening and infiltrating the Nafion film into the Au-NW mesh electrode. On the other hand, higher temperature induces the transformation of the Au-NW (discussed below). For these reasons, hot-pressing temperatures were set between 90 – 130 ºC. IPMC actuator consisting of the Au-foil electrodes was also prepared (Sample #5 in Table 1). The Au-foil (Gold leaf) is an easily accessible material known as “Kinpaku” in Japan, and its thickness is about 100 nm^26^.Figure 1d shows the IPMC actuator strips with the Au-NW mesh and Au-foil electrodes. These are easily distinguishable by the colors of brown and gold.

**Table 1:** Preparation condition of IPMC actuators and physical properties of gold electrodes.

Sample # ^a^	*P *^b^M Pa	*T* ^c^ °C	*d* ^d^ nm	*R*_s_^e^ Ω sq.^−1^
1	3	70	163 ± 21	0.45 ± 0.05
2	3	90	158 ± 29	0.40 ± 0.03
3	3	110	155 ± 36	0.49 ± 0.09
4	3	130	157 ± 25	0.51 ± 0.05
5	3	90	109 ± 27	0.21 ± 0.02

^a^Samples #1–4 and #5 were prepared with Au-NW mesh and Au-foil electrodes, respectively.

^b^ Pressure of hot-pressing.

^c^Temperature of hot-pressing.

^d^Thickness of electrode (Average ± standard deviation, *n* = 10).

^e^Surface resistivity of electrode (Average ± standard deviation, *n* = 5).

### Characterization of Au-NW mesh electrode: effects of heat and pressure on Au-NWs

**Figure 2:**
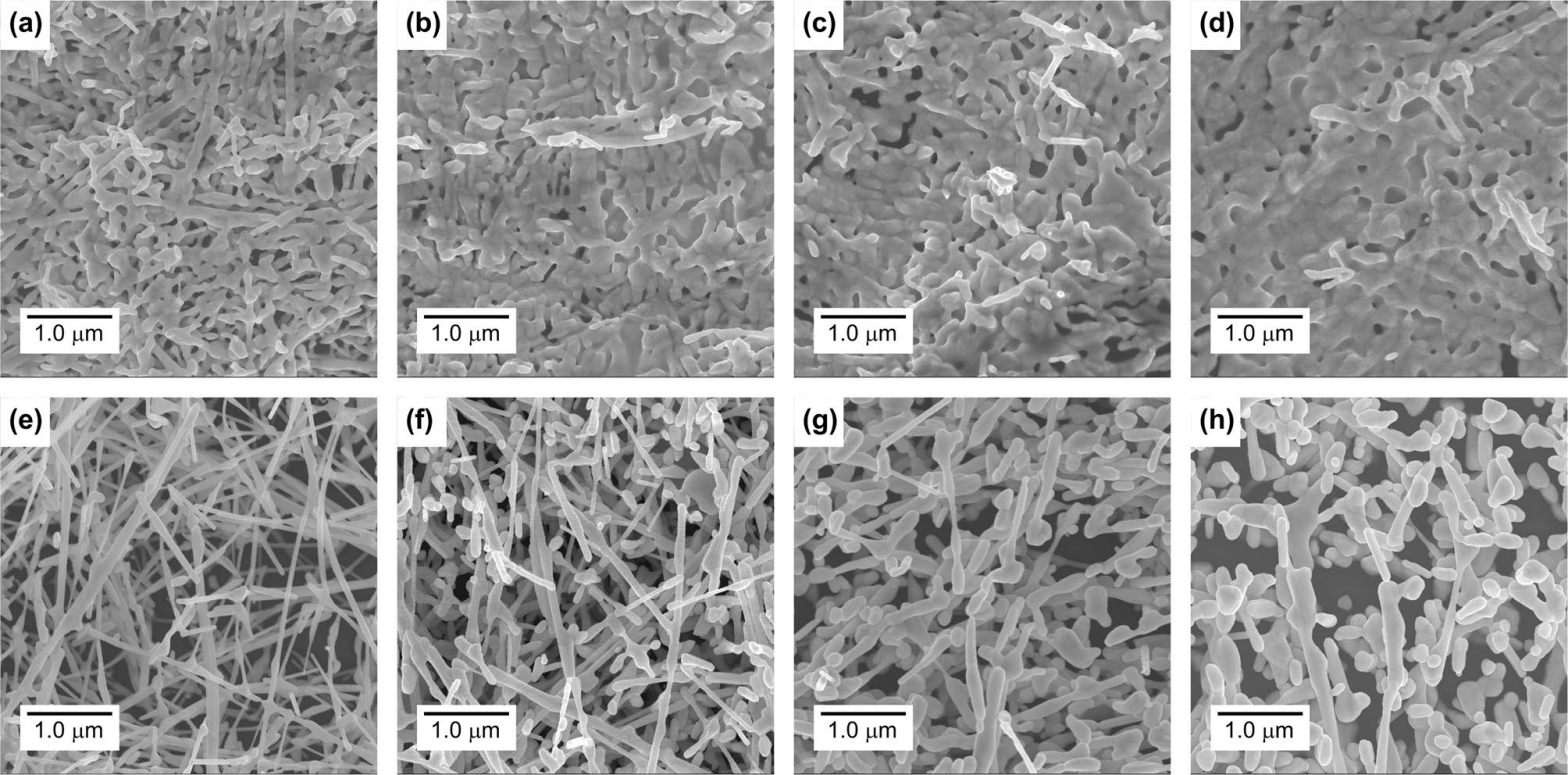
SEM images for examining the effects of hot-pressing on Au-NWs. Conditions: Temperature, Pressure (**a**) 70 °C, 3 MPa, (**b**) 90 °C, 3 MPa, (**c**) 110 °C, 3 MPa, (**d**) 130 °C, 3 MPa, (**e**) 70 °C, no pressure, (**f**) 90 °C, no pressure, (**g**) 110 °C, no pressure, (**h**) 130 °C, no pressure.

Figure 2a–d show the SEM images of IPMC actuator surfaces (sample #1–#4), which correspond to the Au-NW mesh electrodes at the contact part to the Nafion surface in hot-pressing. The definitions of the contact and non-contact part can be referred in Fig. S4. It was confirmed that Au-NWs deformed and transformed to the other shapes by the effects of heat and pressure. Figure 2e–h show the SEM images of Au-NW mesh films at the non-contact part to the Nafion surface. At the non-contact part, the Au-NWs transformed by the effect of heat only (no pressure). The transformation of Au-NWs became significant above 90 °C and more and more Au-NWs transformed to the nano-*particles* with increasing temperature^27^. At the contact part, more and more Au-NWs transformed to the *plates* with increasing temperature. It is interesting to note that the transformation behavior of Au-NWs differs depending on the external stimuli condition.

**Figure 3:**
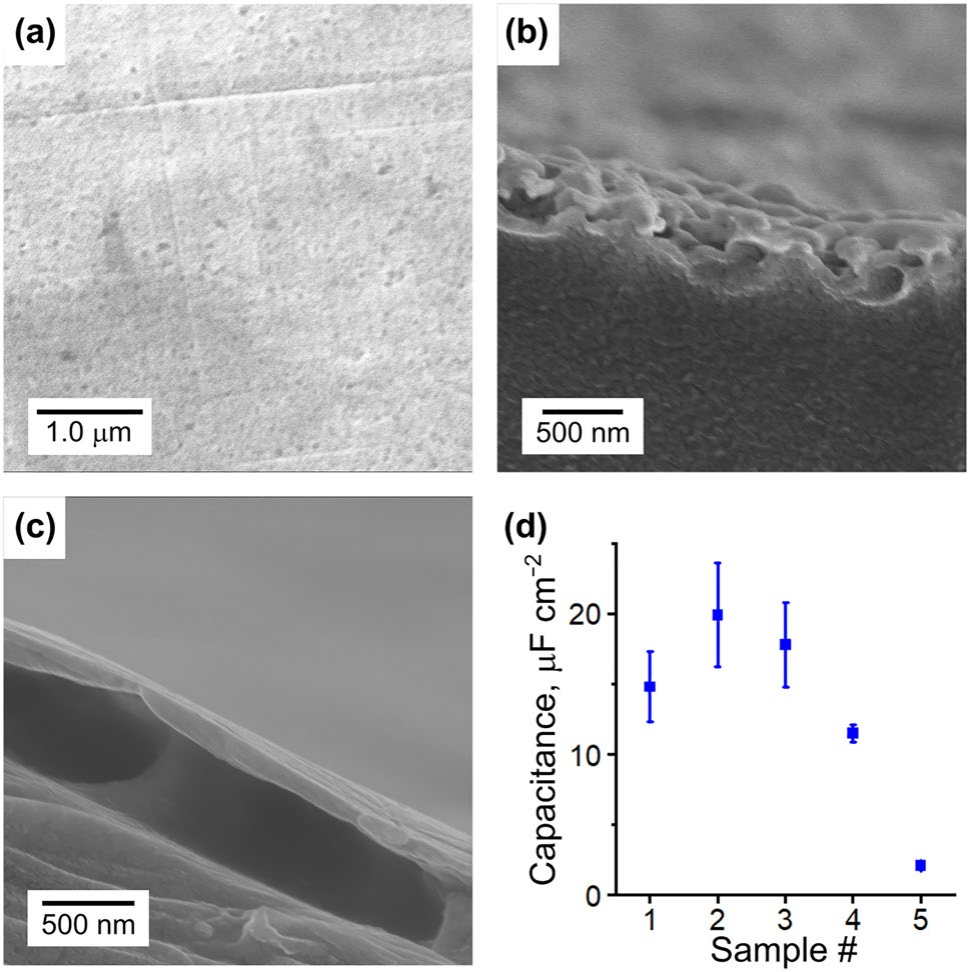
Characterization of surface roughness of gold electrodes. (**a**) SEM image of Au-foil electrode surface (Sample #5). (**b**) SEM image of interface between Nafion and Au-NW mesh electrode (Sample #2). (**c**) SEM image of interface between Nafion and Au-foil electrode (Sample #5). (**d**) Capacitance data of IPMC actuators (Average ± standard deviation, *n* = 5). Data was obtained from impedance data at 0.25 Hz.

The Au-foil electrode has a flat surface (Fig. 3a). Figure 3b,c show the interfaces of the Au-NW mesh and the Au-foil electrodes, respectively. These images indicated that the surface roughness of the Au-NW mesh electrode was larger than that of the Au-foil electrode. Thicknesses of the Au-NW mesh and Au-foil electrodes were *ca.* 160 nm and *ca.* 110 nm, respectively (*d* in Table 1). Compared to the thickness of the electrode prepared by electroless plating (1–10 μm)^3,7,12^, the Au-NW mesh electrode was much thinner. The surface resistivities of Au-NW mesh electrodes (*R*_s_ in Table 1) were lower than that of Au-NW mesh film (1.23 ± 0.24 Ω sq^−1^) because of the improved connectivity between the Au-NWs by hot-pressing. It should be noted that the surface resistivity of Au-NW mesh electrode (0.4–0.5 Ω sq^−1^) is much lower than that of the electrode prepared by electroless plating (1–10 Ω sq^−1^) despite of its smaller thickness^7,12,28^. The surface resistivity of the Au-foil (0.21 ± 0.02 Ω sq^−1^) was lower than that of the Au-NW mesh electrode.

Since the contact surface area of the electrode to the Nafion surface is a key parameter to determine the IPMC actuator performance^12,29,30^, the capacitance was measured. The capacitance of the IPMC with the Au-foil electrodes (Sample #5) was much lower than those with the Au-NW mesh electrodes (Fig. 3d). It is considered that rough surface of the Au-NW mesh electrode contributes to increase the contact surface area to the Nafion surface^18^. Among the IPMCs with the Au-NW mesh electrodes, the sample #2 had the highest capacitance. While higher temperature is advantage to improve the adhesion of the Au-NW mesh electrode to the Nafion surface, it induces the transformation of the nanowire to the plates. It is considered that the transformation of the Au-NWs to the plates decreases the surface roughness (contact area) of the electrode. As a consequence, the preparation temperature of 90 °C was the best to get high capacitance.

### Characterization of actuation

**Figure 4:**
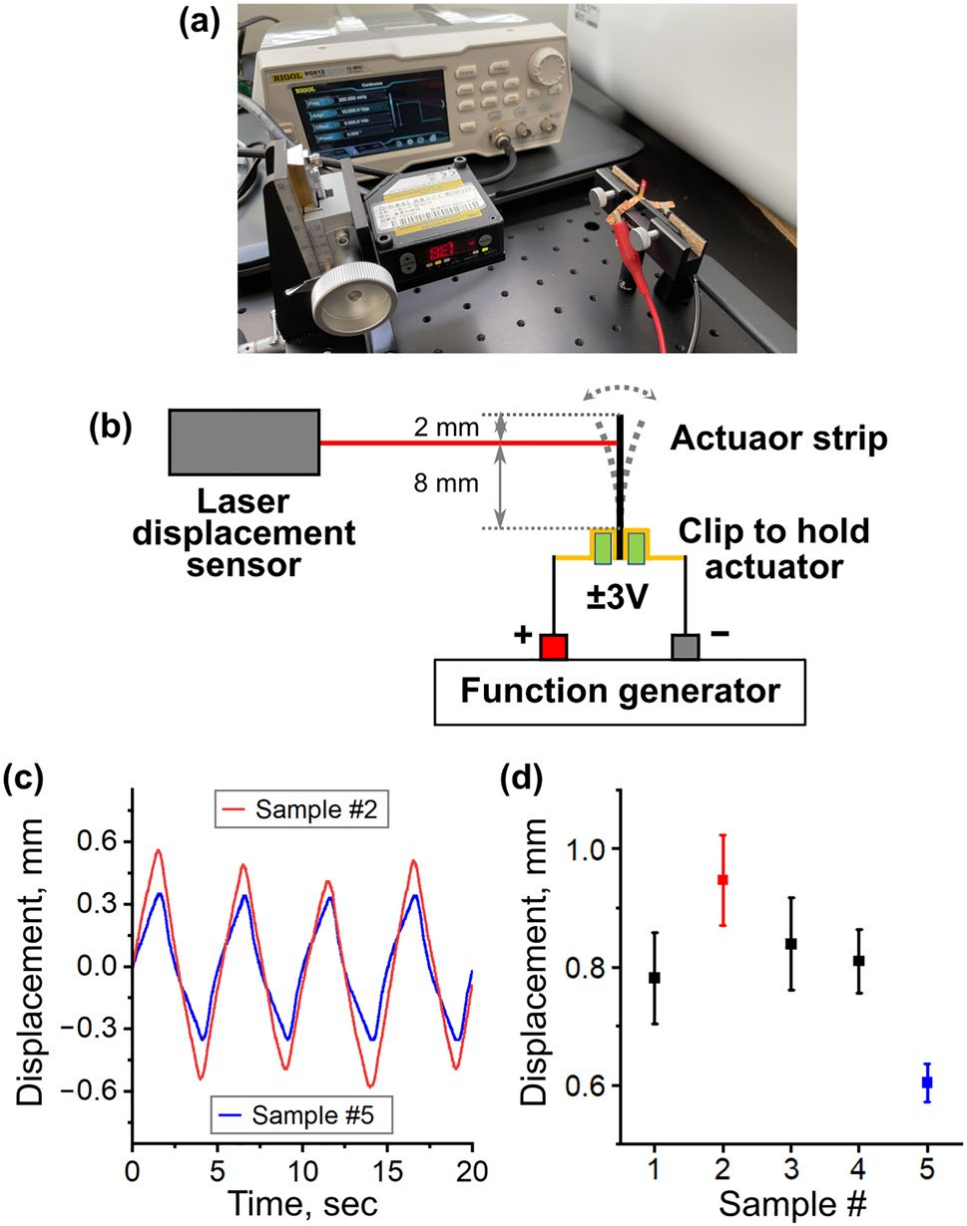
Characterization of actuation behavior. (**a**) Photograph of experimental setup of actuation measurement. (**b**) Schematic illustration of experimental setup of actuation measurement. (**c**) Actuation behaviors of IPMC actuators performed under ± 3 V square wave voltage with the frequency of 0.2 Hz. Red wave: Sample #2. Blue wave: Sample #5. (**d**) Peak-to-peak displacements of IPMC actuators measured under the same condition as (**c**) (Average ± standard deviation, *n* = 5).

The actuation behavior was characterized using a laser displacement sensor (Fig. 4a,b). When the square-wave voltage of ± 3 V was applied with the frequency of 0.2 Hz, the bending motion of the actuator strip was monitored. The representative results are shown in Fig. 4c. It was confirmed that the IPMC actuator with the Au-NW mesh electrodes (Red wave: sample #2) showed larger displacement than that with the Au-foil electrodes (Blue wave: sample #5). Figure 4d summarizes the peak-to-peak displacements of all samples. It was confirmed that all IPMC actuators with the Au-NW mesh electrodes gave larger displacement than that with the Au-foil electrodes. Among the IPMC actuators with the Au-NW mesh electrodes, the sample #2 exhibited the best performance, which is consistent to the results of the capacitance measurement. The bending mechanism of the IPMC actuators has been proposed that the hydrated cations move toward the cathode inside the Nafion film under the applied potential, which results in the volume expansion of Nafion at the cathode side^2^. Based on this mechanism, it is considered that the IPMC actuator possessing higher capacitance can collect more hydrated cations on the cathode and induce larger deformation. No detachment of the Au-NW mesh electrode from the Nafion surface was observed during the measurements.

## Conclusion

IPMC actuators consisting of Nafion and Au-NW mesh electrodes were successfully prepared. The performance of the IPMC actuators with Au-NW mesh electrodes is superior to that with Au-foil electrode because of larger contact area between the electrode and the Nafion surface. The transformation behavior of Au-NWs differed depending on the external stimuli condition. Namely the Au-NWs transformed to the Au nanoparticles in the case of the heat stimulus only. Meanwhile, the Au-NWs transformed to the plates in the case of heat and pressure stimuli. While higher temperature improved the adhesion of Au-NW mesh electrode to the Nafion surface, it induced the transformation of nanowire to plates. The IPMC actuator that the Au-NW mesh electrodes were hot-pressed at 90 ºC exhibited the highest capacitance and the largest peak-to-peak displacement in actuation. It takes some days to prepare the Au-NWs and their mesh films. However, Au-NW mesh films can be stored at room temperature in air under dark. Utilizing the stock Au-NW mesh films, one can prepare the IPMC actuators within one hour. It is a big advantage to the conventional preparation process with electroless plating. Since Au-NW mesh film can be the electrode with thinner and better electrical conductivity than that prepared by electroless plating, the Au-NW mesh electrode is a promising material for electromechanical devices. As a direction for future research, it is desirable to develop a new preparation process to attach the Au-NW mesh electrodes on the Nafion film without inducing the morphology change of Au-NWs. If Nafion could be infiltrated into Au-NW mesh electrodes more deeply, the improvement of the device performance could be expected, which will pave the way for the IPMC actuators to install in the biomedical devices, flexible robots and microelectromechanical systems.

### Supplementary Information

Supplementary Information.

## Data Availability

All data generated or analyzed during this study are included in this published article [and its supplementary information files].
