# Supplementary material for: Gold nanowire mesh electrode for electromechanical device
Source: Sci Rep. 2023 Oct 4;13:16669. doi: 10.1038/s41598-023-43960-8 (PMC10551032; doi:10.1038/s41598-023-43960-8)
Supplement: Supplementary file 1 — Supplementary Information. [file 41598_2023_43960_MOESM1_ESM.pdf]

Supplementary material

Gold Nanowire Mesh Electrode for Electromechanical Device

Taichi Ikeda*

Research Center for Macromolecules and Biomaterials,
National Institute for Materials Science,
1-1 Namiki Tsukuba Ibaraki, 305-0044, Japan
E-mail: IKEDA.Taichi@nims.go.jp

Table of contents

1. **Characterization of gold nanowire** S2
2. **Experimental setup of IPMC actuator preparation** S3
3. **Summary of capacitance data** S4
4. **Summary of actuation data** S4

1. Characterization of gold nanowire

The length of the gold nanowire was obtained from the SEM image of the gold nanowires on a HOPG substrate. The suspension of gold nanowires in 0.1 M CTAB aqueous solution was dropped on a fleshly-cleaved HOPG surface. After keeping the sample for 5 min, the solvent was removed by tilting the substrate and capillary action of the paper. The substrate was washed with distilled water to remove CTAB. The length and width of the gold nanowires were measured from the SEM images with the software ImageJ Ver. 1.53k (http://imagej.nih.gov/ij).

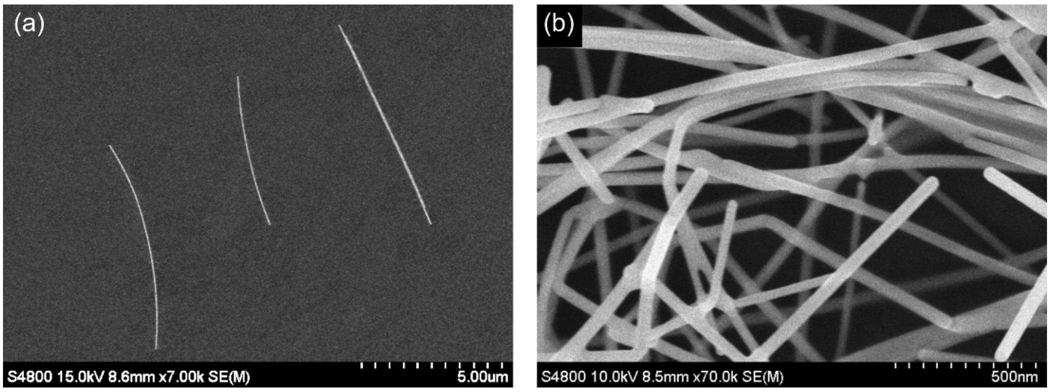

Figure S1. Representative SEM images of (a) gold nanowires on a HOPG surface for length measurement and (b) gold nanowire film for width measurement.

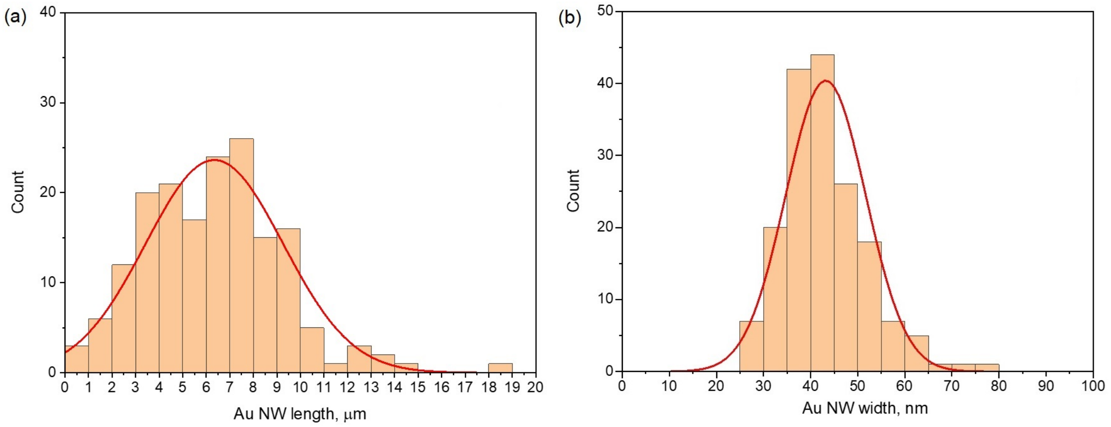

Figure S2. (a) Histograms showing size distribution of gold nanowires. (a) length (6.3 ± 2.9 μm) and (b) width (43.2 ± 8.5 nm).

2. Experimental setup of IPMC actuator preparation

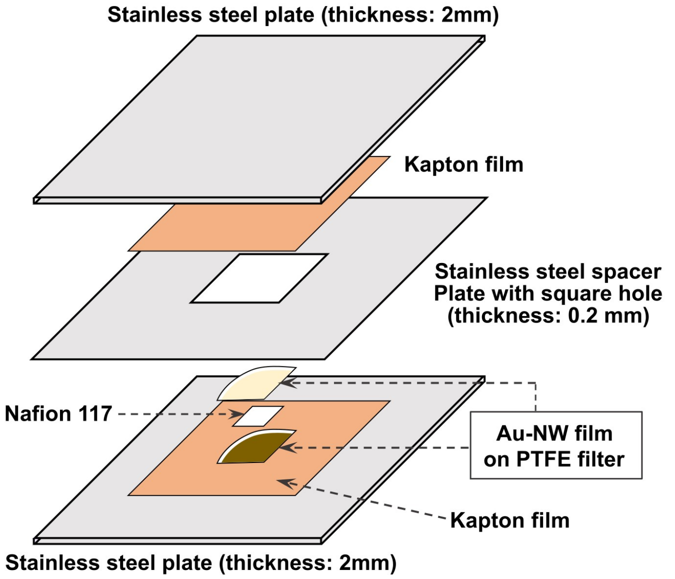

Figure S3 Schematic illustration of the assembled plates for preparing IPMC actuators by hot pressing.

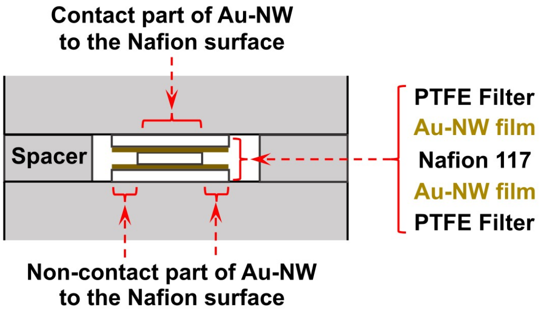

Figure S4 Definition of contact and non-contact parts of Au-NW film to the Nafion surface in hot pressing process.

3. Summary of capacitance data

Table. S1 Capacitance data of IPMC

	Sample #1 F cm^{-2}	Sample #2 F cm^{-2}	Sample #3 F cm^{-2}	Sample #4 F cm^{-2}	Sample #5 F cm^{-2}
1	1.20E−5	2.52E−5	2.09E−5	1.20E−5	2.13E−6
2	1.81E−5	1.87E−5	1.43E−5	1.06E−5	2.22E−6
3	1.51E−5	1.93E−5	2.06E−5	1.17E−5	2.06E−6
4	1.27E−5	2.11E−5	1.80E−5	1.13E−5	2.08E−6
5	1.62E−5	1.50E−5	1.54E−5	1.20E−5	2.06E−6
Average	1.48E−5	1.99E−5	1.78E−5	1.15E−5	2.11E−6
STD	2.48E−6	3.73E−6	2.99E−6	6.09E−7	6.90E−8

4. Summary of actuation data

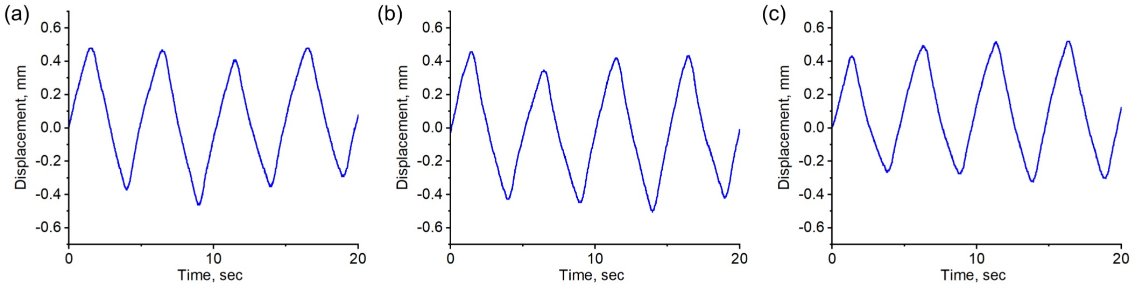

Figure S5 Actuation behaviors of IPMC actuators (±3V square wave voltage, 0.2 Hz frequency). (a) Sample #1, (b) Sample #3, (c) Sample #4.

Table S2 Peak-to-peak displacements of IPMC actuators. (±3V square wave voltage, 0.2 Hz frequency)

	Sample #1 mm	Sample #2 mm	Sample #3 mm	Sample #4 mm	Sample #5 mm
1	0.80	1.03	0.83	0.77	0.61
2	0.87	1.02	0.97	0.86	0.64
3	0.69	0.92	0.76	0.85	0.61
4	0.72	0.84	0.81	0.74	0.62
5	0.83	0.39	0.83	0.83	0.55
Average	0.78	0.95	0.84	0.81	0.60
STD	0.08	0.08	0.08	0.05	0.03
